# Supplementary material for: DEFECTIVELY ORGANIZED TRIBUTARIES 5 is not required for leaf venation patterning in Arabidopsis thaliana
Source: Plant J. 2022 Sep 13;112(2):451–9. doi: 10.1111/tpj.15958 (PMC9826136; doi:10.1111/tpj.15958)

**SUPPORTING INFORMATION – Vlad & Langdale**

**Figure S1. Validation of the T-DNA insertion at the *AtDOT5* locus in the homozygous SALK_148869c line. A)** Schematic representation of the T-DNA insertion at the *AtDOT5* locus (SALK_148869.53.50.x). Black boxes depict exons with the overlying red box depicting the WIP domain and the blue boxes depicting the zinc finger domains. Inverted triangle marks the position of the T-DNA in the first exon and the red arrows show the positioning of the genotyping primers. **B)** PCR validation of T-DNA insertion at the *AtDOT5* locus (At1g13290). Primers were designed in the T-DNA border (LBa1: 5’-TGGTTCACGTAGTGGGCCATCG-3’) and in the first exon of *AtDOT5* both upstream (LP: 5’-TTCCCACAATTTTTCTCATGC-3’) and downstream (RP: 5’-CATGGGCTTGTCACTCGTAAC -3’) of the T-DNA insertion. The expected 438-738 bp fragment was amplified by PCR using LBa and RP primers. Amplification using the LP and RP primers yielded the expected 1062 bp fragment in Col-0 but no fragments in the SALK_148869c line due to the presence of the T-DNA insertion. The SALK_148869c line is thus homozygous for the T-DNA insertion at *AtDOT5*. **C)** PCR showing absence of a T-DNA insertion at the soluble epoxide hydrolase encoding At2g26740 locus. Because an additional insertion (SALK_148869.13.90.x) was reported in the parental line SALK_148869, primers were designed to amplify sequences from the At2g26740 locus (in an analogous design to that for *AtDOT5*) in the SALK_148869c line. No fragments were amplified using the LBa1 and RP (5’- TCAATTTGGTTAATGATTTGCC-3’) primers, demonstrating the absence of a T-DNA insertion at the locus. By contrast, a 1172 bp fragment was amplified from all SALK-148869c individuals and from Col-0 using RP and LP (5’-CCCCAAAAACTTTTGTCCTTC-3’) primers.

**
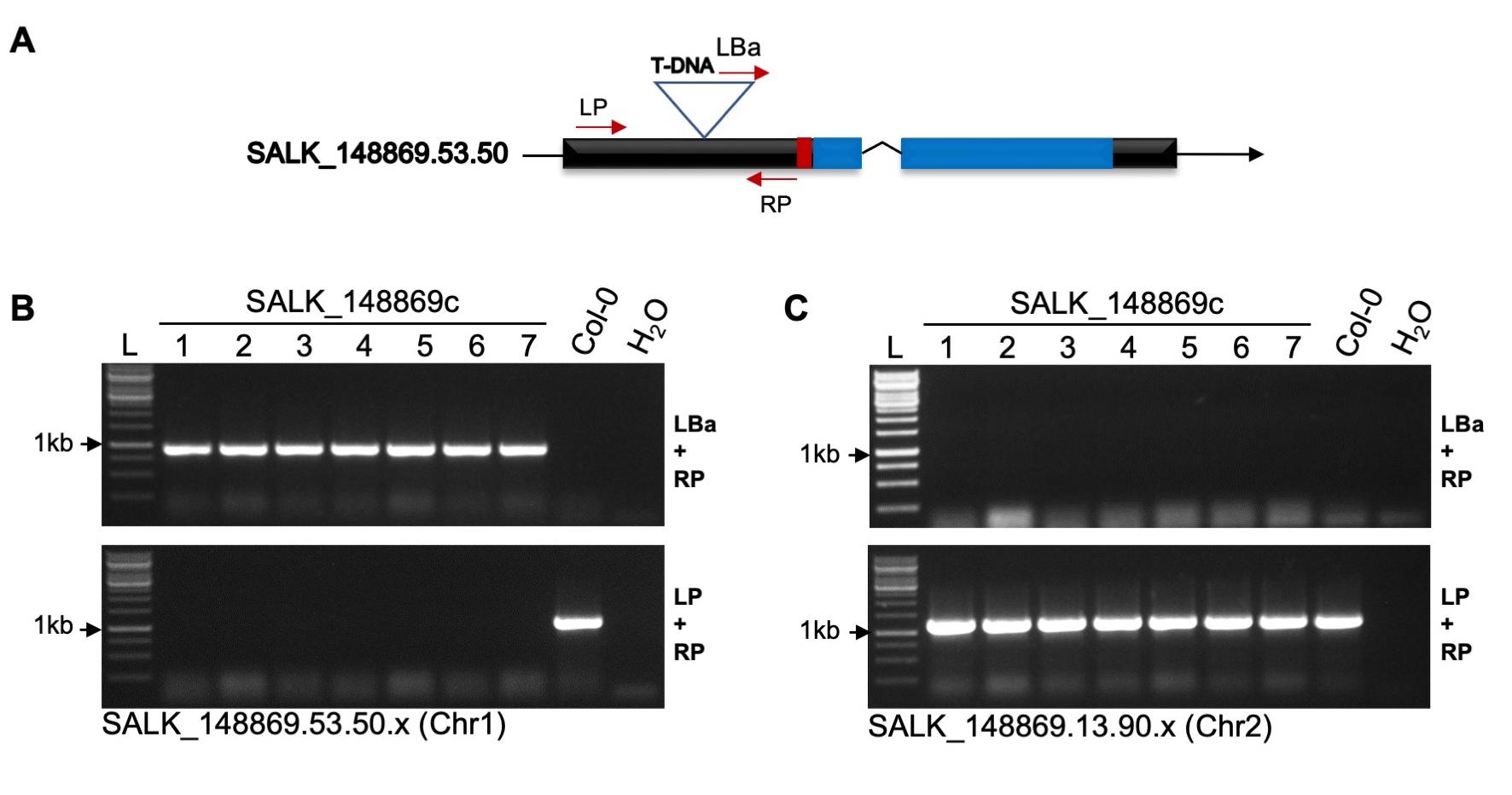
**

**Figure S2. Snapshots of the sequencing reads for ten genes flanking either side of *AtDOT5,* aligned to the Landsberg *erecta* reference genome**. Reads were visualized using the Integrative Genomics Viewer (IGV) software. Reads are shown as arrows, the blue bars at the bottom of the image are gene models with wider regions representing exons.

**ATLER-1G23870**


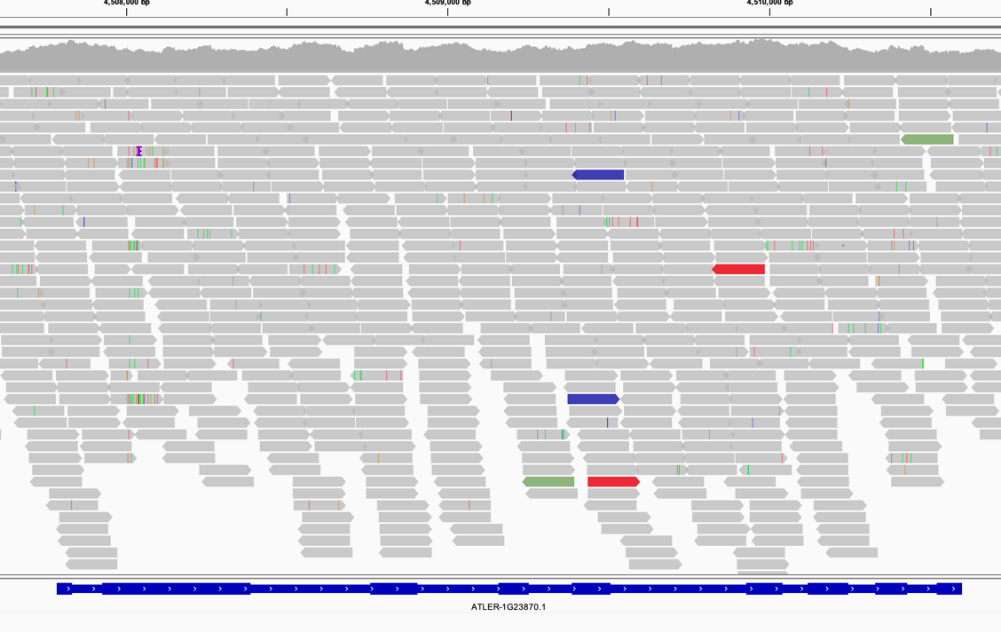


**ATLER-1G23880 and ATLER-1G23890**


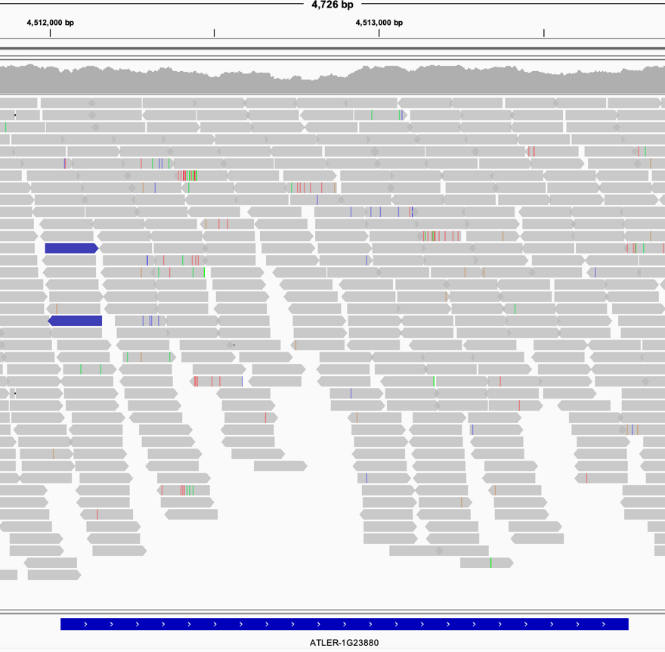

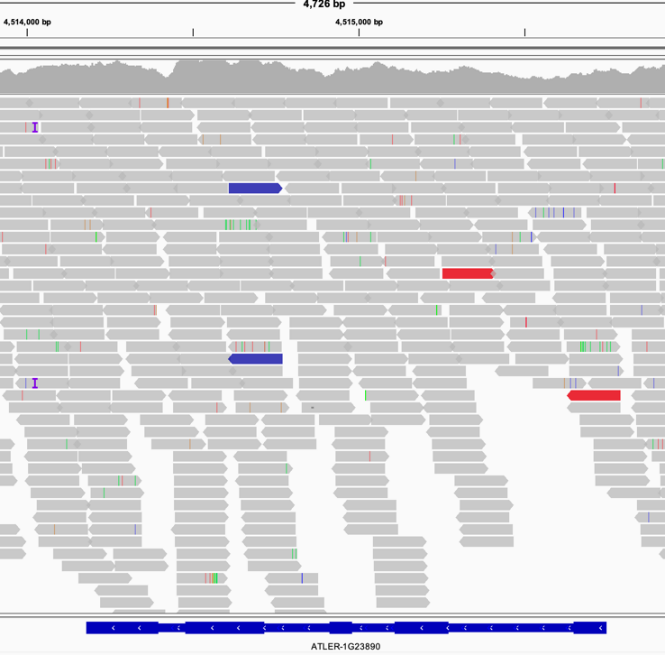


**ATLER-1G23900**


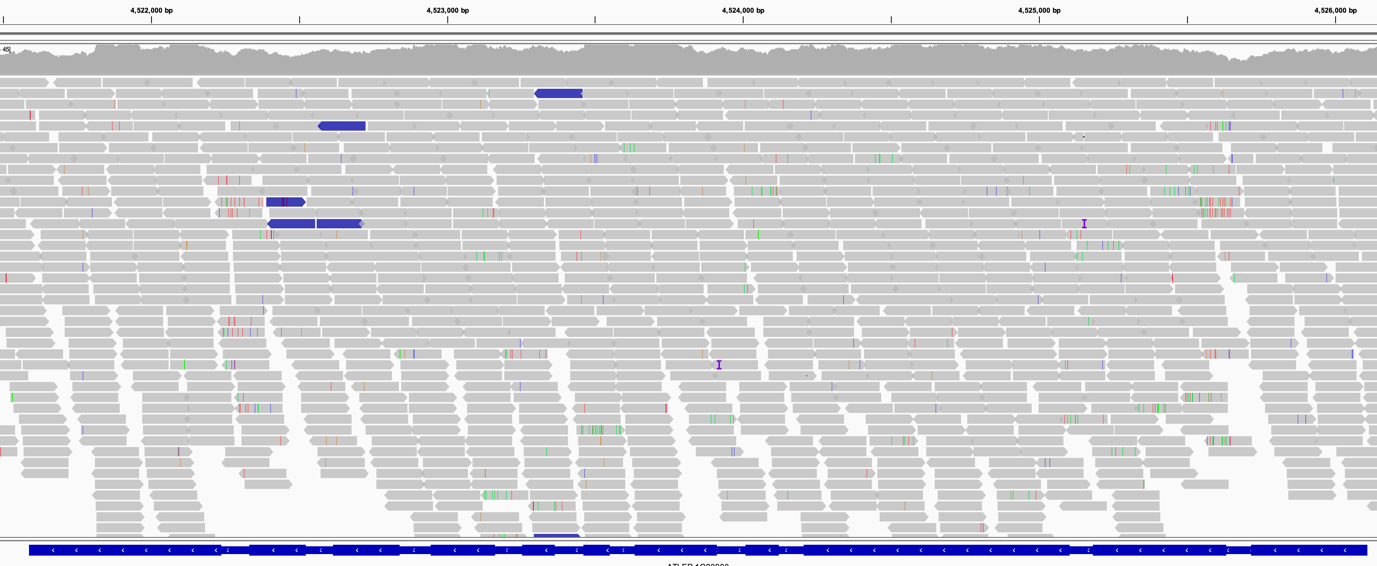


**ATLER-1G23910**


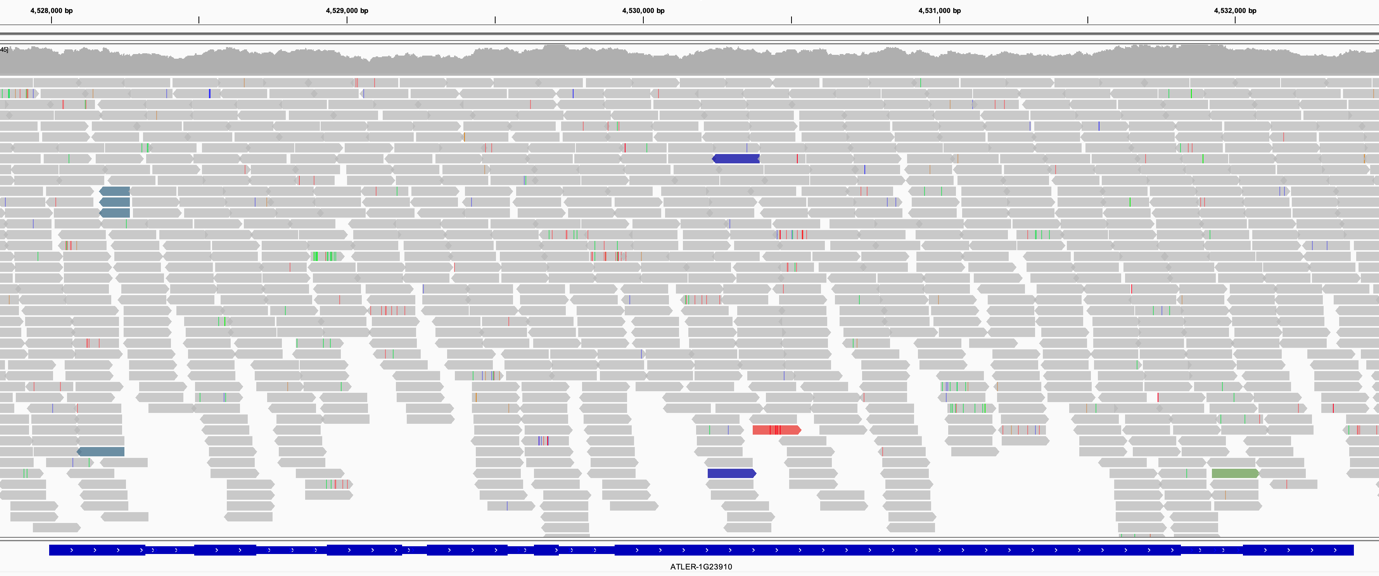


**ATLER-1G23920**


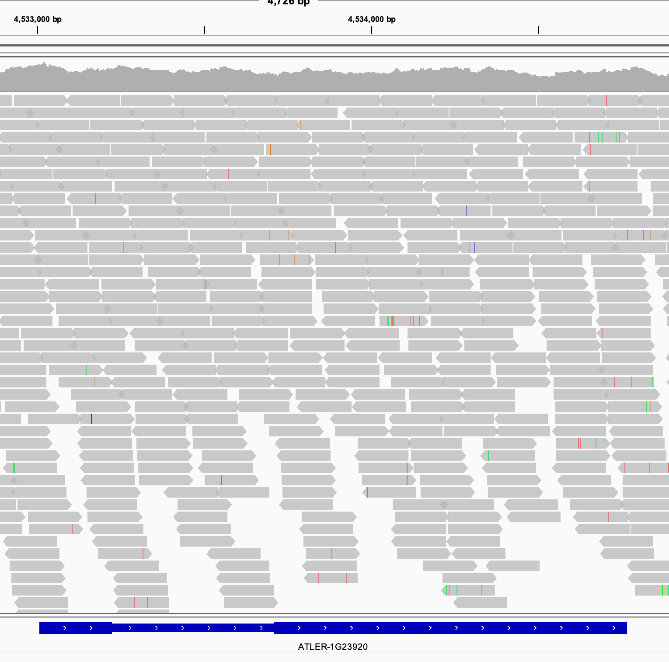


**ATLER-1G23940**


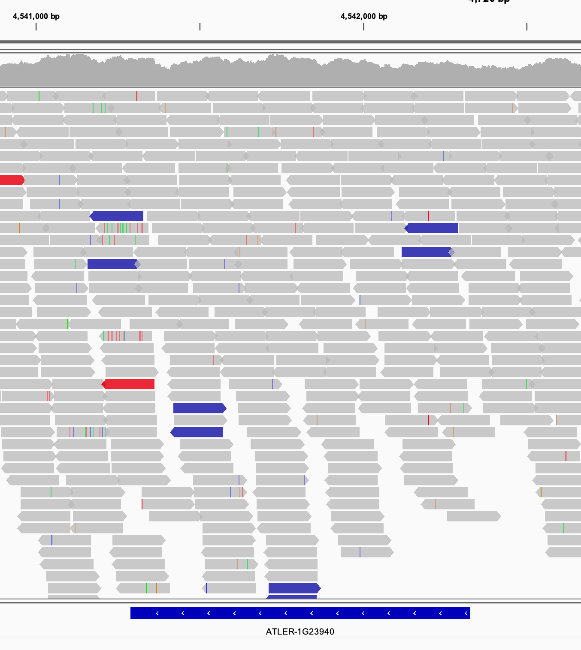


**ATLER-1G23950**


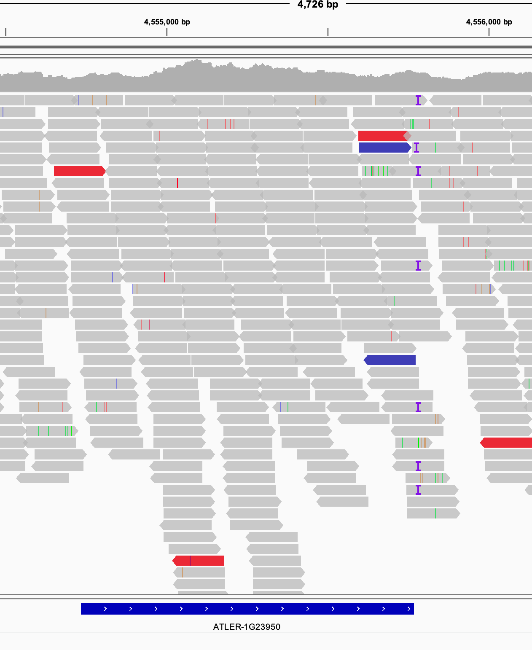


**ATLER-1G23960**


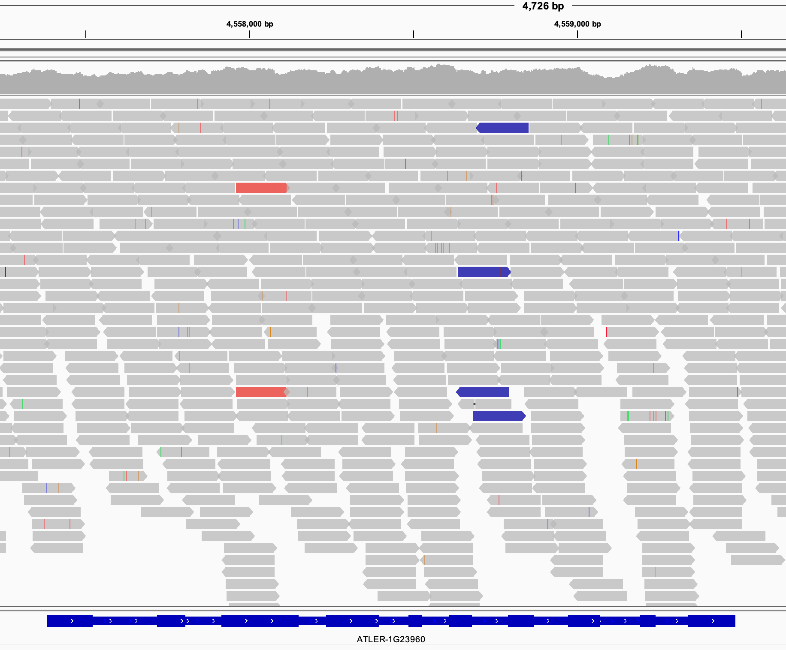


**ATLER-1G23970**


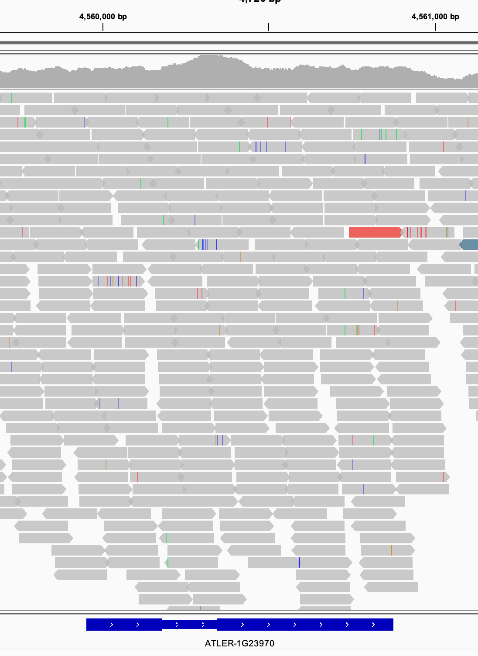


**ATLER-1G23980 (DOT5)**


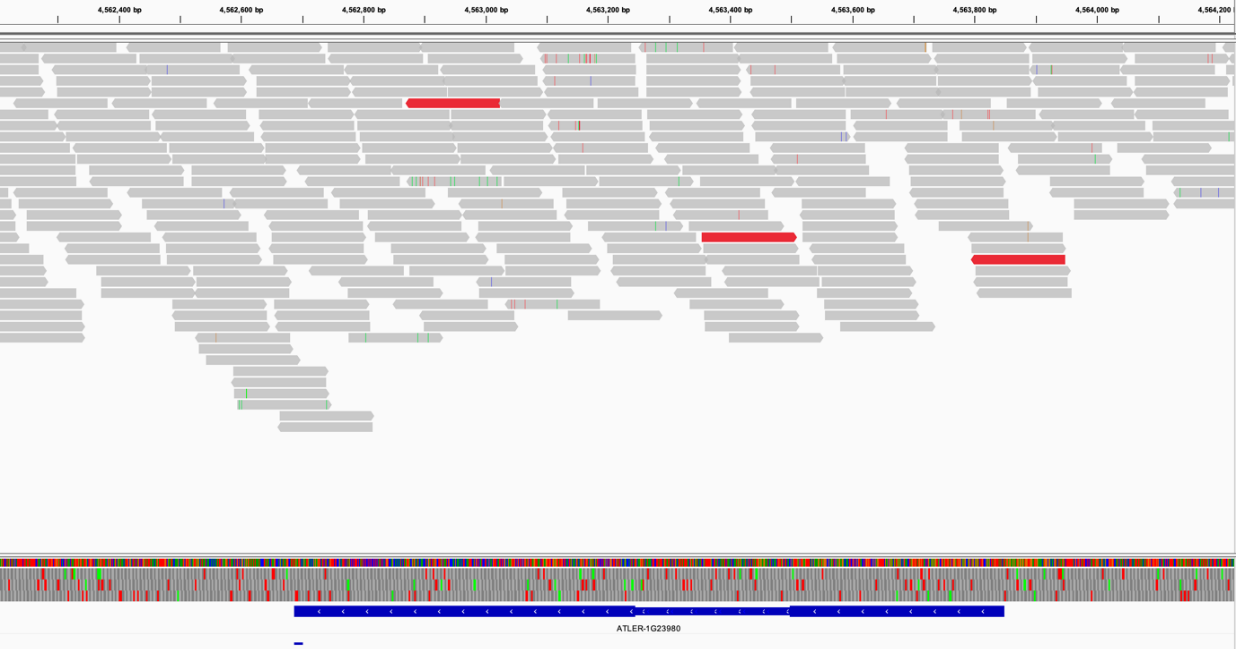


**ATLER-1G23990**


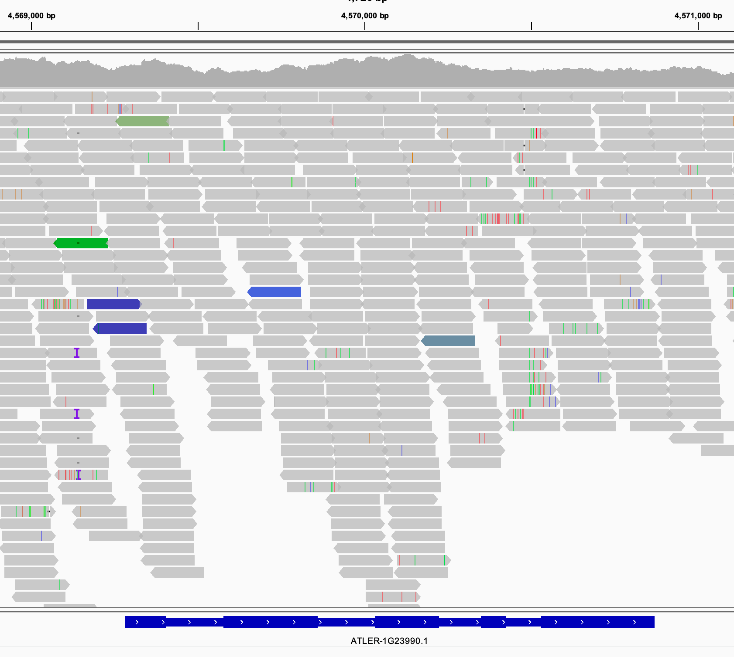


**ATLER-1G24000**


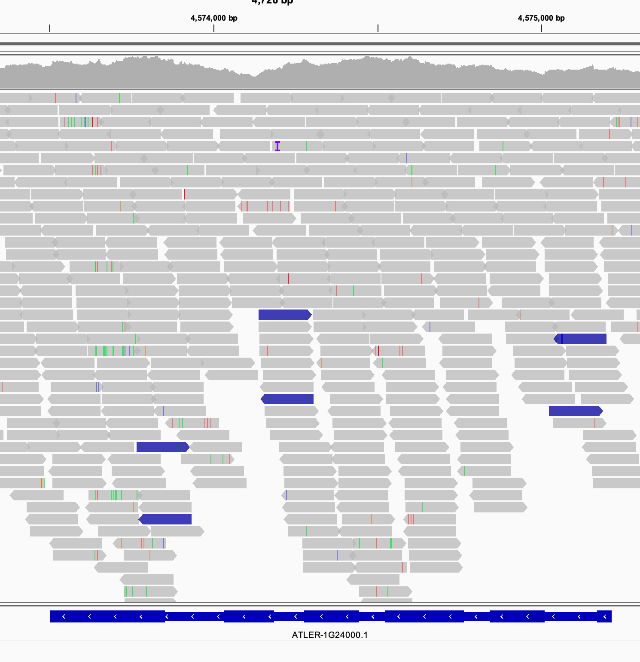


**ATLER-1G24010**


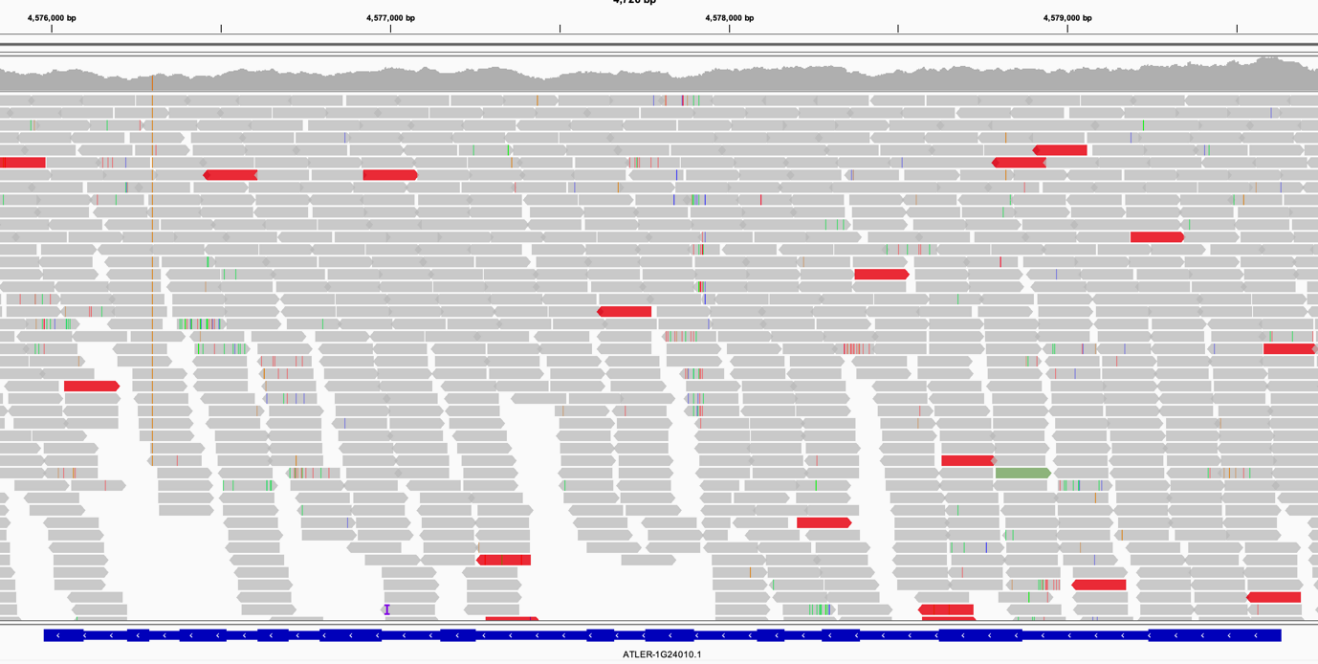


**ATLER-1G24020 and ATLER-1G24030**


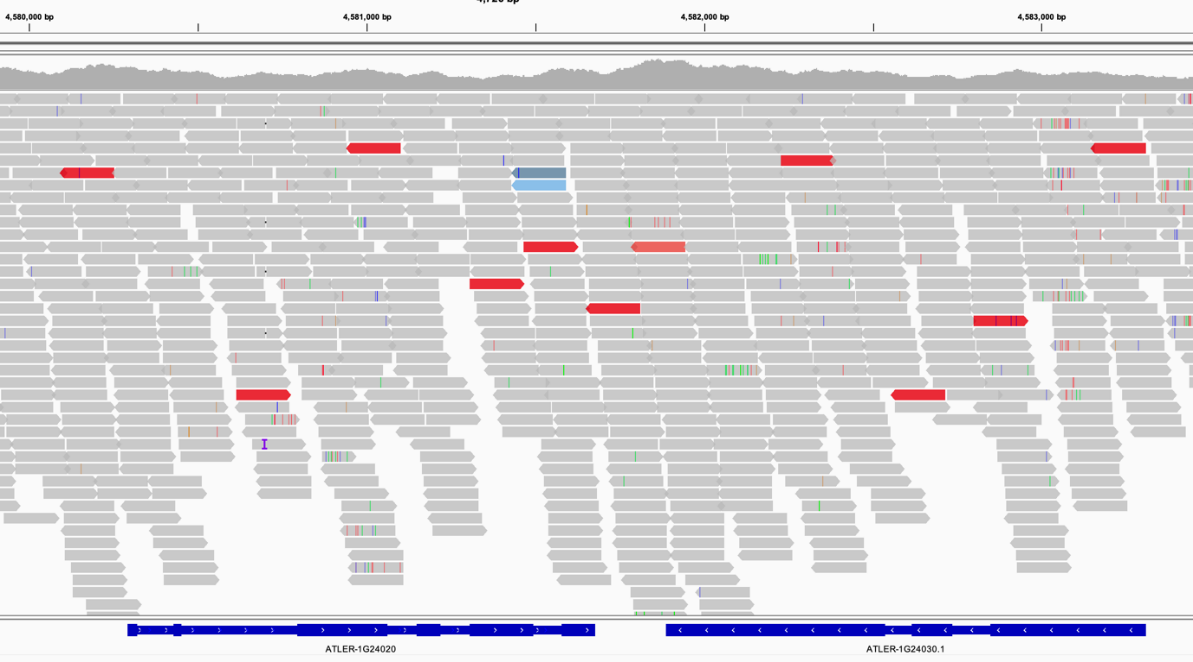


**ATLER-1G24040**


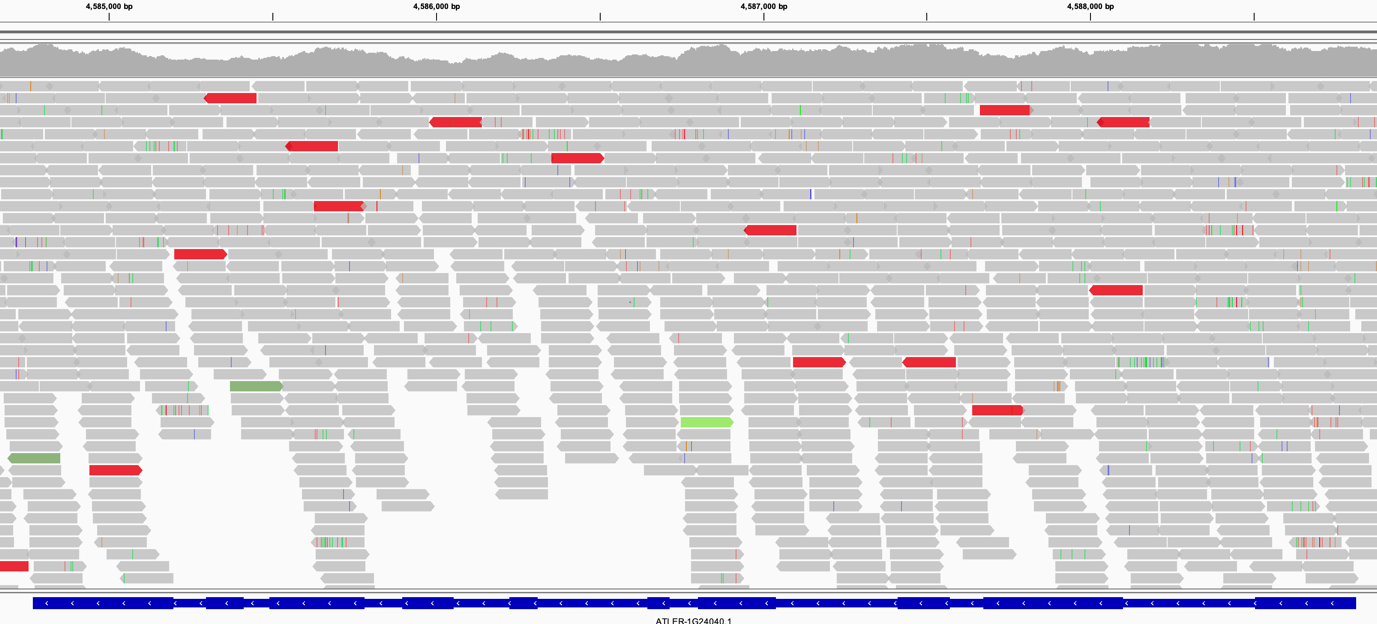


**ATLER-1G24050 and ATLER-1G24060**


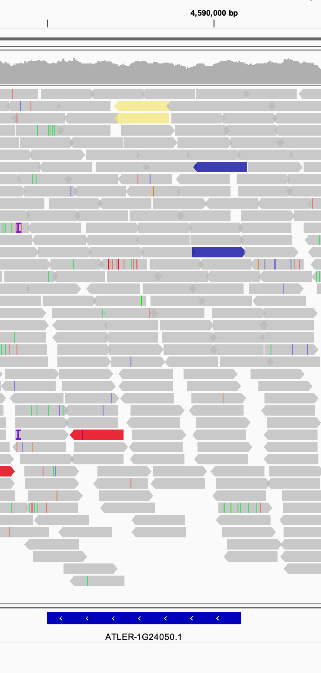

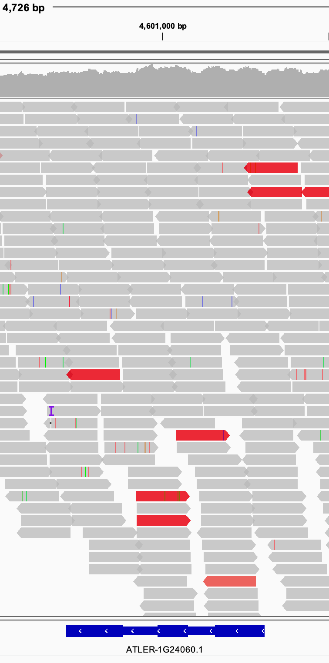


**ATLER-1G24070**


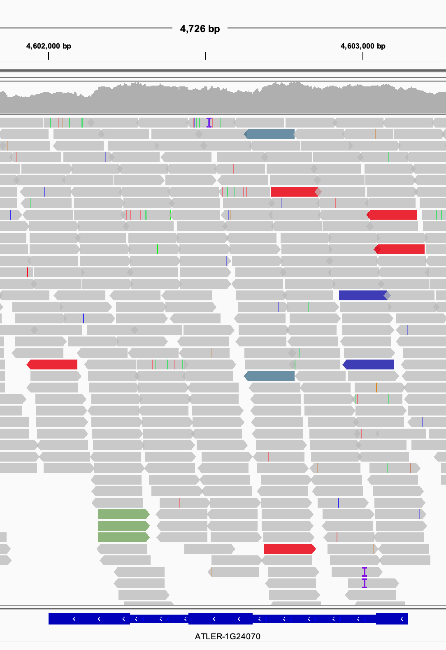


**ATLER-1G24080**


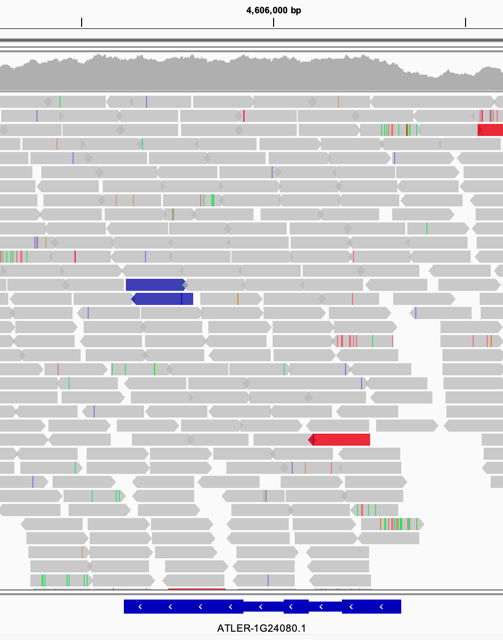


**Figure S3.** **Confirmation that the *Ds-*containing transgene insertion is in the same position in *Atdrl1-1* and *Atdot5-1* mutants. A)** Schematic representation of the *Ds* insertion in the *DRL1* promoter region. Inverted triangle marks the position of the insertion and the red arrows show the position of the forward (F) and reverse (R) genotyping primers used for PCR. **B)** PCR amplicons of similar sizes were obtained from both the *Atdrl1-1* and *Atdot5-1* mutants suggesting that the insertion is present at the same location in both mutant backgrounds.

**
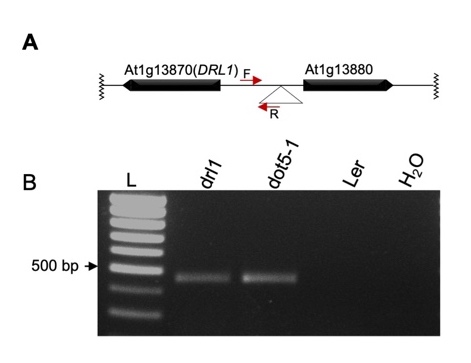
**

**Figure S4. Genotyping of the *AtDOT5* CRISPR alleles**. **A)** Sequence containing the first exon showing the position of the guide sequence (reverse) highlighted in blue with the PAM site shown in italics and the restriction site for *RsaI* (GTAC) underlined. Cas9 will induce mutations next to the PAM. If a mutation is induced, *RsaI* is unlikely to cut the PCR product amplified from the mutant sequence. Primers are highlighted in green. **B)** Example of an *RsaI* digest of fragments amplified from a segregating T3 population (samples 1 to 18). The lower fully digested band is amplified from the wild type allele and the upper undigested band from the mutant allele. L indicates the molecular weight marker.


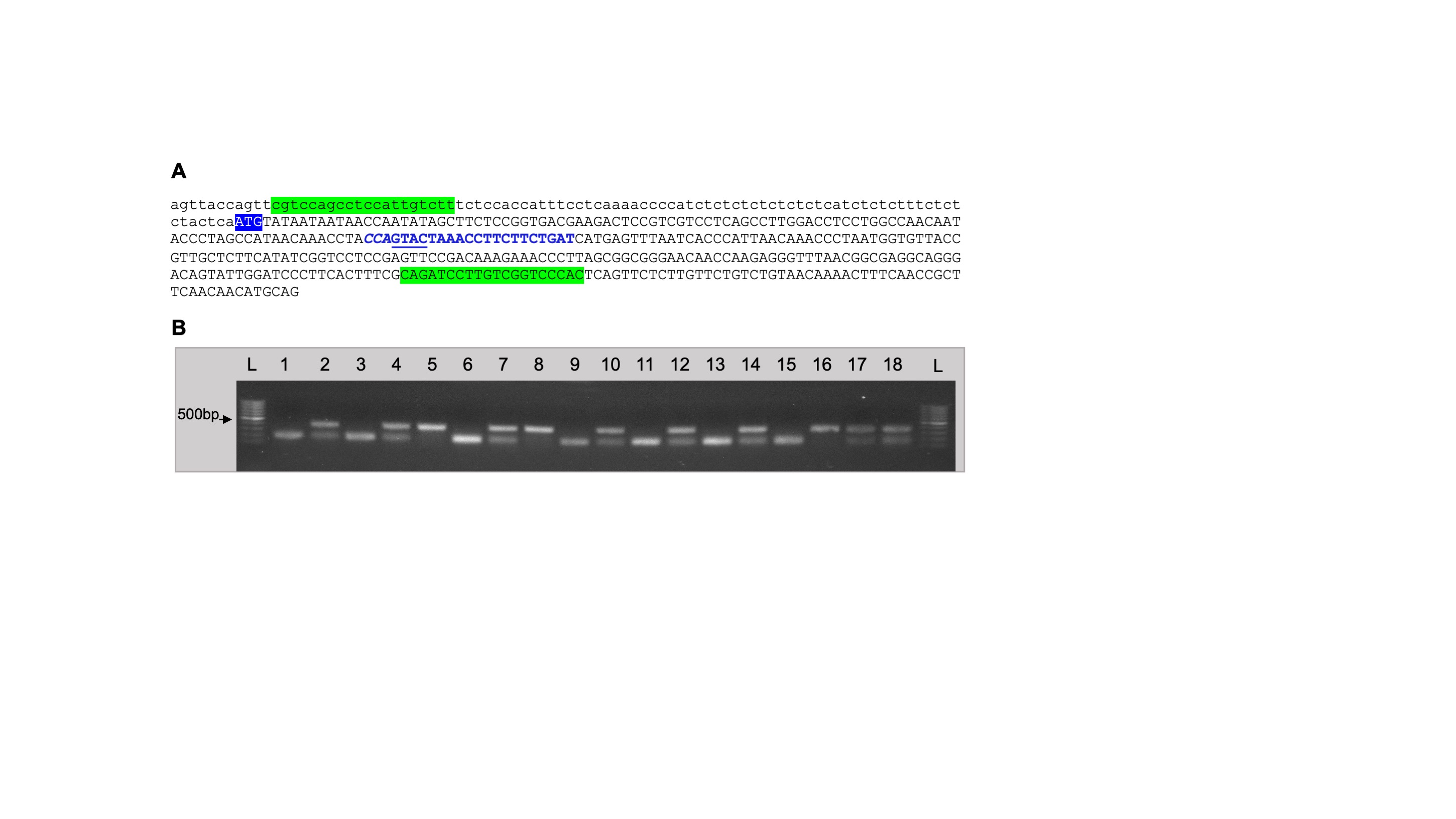

Supplement: Supplementary file 1 — Figure S1. Validation of T‐DNA insertion in AtDOT5 in the SALK_148869c line. Figure S2. Snapshots of sequencing reads for genes flanking AtDOT5 in the Atdot5‐1 genome. Figure S3. Confirmation of shared transgene position in Atdot5‐1 and Atdrl1‐1 mutants. Figure S4. PCR assay to genotype gene edited alleles of AtDOT5. [file TPJ-112-451-s001.docx]
